# CT and MR Imaging of Retroperitoneal Sarcomas: A Practical Guide for the Radiologist

**DOI:** 10.3390/cancers15112985

**Published:** 2023-05-30

**Authors:** Giorgia Porrello, Roberto Cannella, Angelo Randazzo, Giuseppe Badalamenti, Giuseppe Brancatelli, Federica Vernuccio

**Affiliations:** 1 Section of Radiology, Department of Biomedicine, Neuroscience and Advanced Diagnosis (Bi.N.D), University of Palermo, 90127 Palermo, Italy; giorgia.porrello@gmail.com (G.P.); rob.cannella89@gmail.com (R.C.); gbranca@yahoo.com (G.B.); 2 Radiology Unit, Department of Diagnostic and Therapeutic Services, IRCCS ISMETT (Mediterranean Institute for Transplantation and Advanced Specialized Therapies), Via Tricomi 5, 90127 Palermo, Italy; 3 Department of Radiology, Azienda Sanitaria Provinciale, 92100 Agrigento, Italy; angelo.randazzo.90@gmail.com; 4 Section of Medical Oncology, Department of Surgical, Oncological and Oral Sciences (DICHIRONS), University of Palermo, 90127 Palermo, Italy; giuseppe.badalamenti@unipa.it; 5 Department of Radiology, University Hospital of Padova, 35128 Padova, Italy

**Keywords:** sarcoma, retroperitoneal neoplasms, magnetic resonance imaging, computed tomography, liposarcoma, leiomyoma, solitary fibrous tumor, nerve sheath tumor, differential diagnosis, complications

## Abstract

**Simple Summary:**

The diagnosis of retroperitoneal sarcoma may be challenging for the radiologist. Current guidelines report postsurgical margin as the strongest predictive factor for disease-specific survival and recurrence, as well as histologic subtype and grade. The role of the radiologist is indeed important in RPS diagnosis, management, and follow-up, as the ability to promptly recognize local progression, invasion of nearby structures, and complications has a direct impact on patients’ survival. A practical guide is provided to radiologists with an overview of the current knowledge regarding cross-sectional CT/MRI imaging features of patients with retroperitoneal sarcomas, presenting tips and tricks to improve imaging diagnosis of RPS.

**Abstract:**

Primary retroperitoneal sarcomas (RPS) represent around 10–16% of all sarcomas, with liposarcomas and leiomyosarcomas being the most common subtypes. RPS have some peculiar characteristics, imaging appearances, worse prognosis, and complications compared to other locations of sarcoma. Commonly, RPS primarily present as large masses, progressively encasing adjacent structures, causing mass effect, and complications. RPS diagnosis is often challenging, and these tumors may be overlooked; however, failure to recognize RPS characteristics leads to a worse prognosis for the patients. Surgery is the only recognized curative treatment, but the anatomical constraints of the retroperitoneum limit the ability to achieve wide resection margins; therefore, these tumors have a high rate of recurrence, and require long-term follow-up. The radiologist has an important role in the diagnosis of RPS, the definition of their extent, and their follow-up. Specific knowledge of the main imaging findings is required to reach an early diagnosis, and, ultimately, to guarantee the best patient management. This article provides an overview of the current knowledge regarding cross-sectional imaging features of patients with retroperitoneal sarcomas, presenting tips and tricks to improve imaging diagnosis of RPS.

## 1. Background

Retroperitoneal sarcomas (RPS) are rare and aggressive tumors, with an incidence of 0.5–1 new case per 100,000 inhabitants per year [1,2,3], accounting for 10–16% of all sarcomas [4,5,6,7]. RPS definition encompasses a heterogeneous and complex group of neoplasms, with over 60 different histological subtypes, each one with different biological behavior, response to treatment, and different oncologic survival [8]. Among them, four types represent about 90% of all cases: liposarcoma (LPS), leiomyosarcoma (LMS), solitary fibrous tumor (SFT) and malignant peripheral nerve sheath tumor (MPNST) [1], with well-differentiated/dedifferentiated liposarcomas (40–70%) and leiomyosarcomas (27%) being the most common subtypes [5,6,9].

The retroperitoneum is a particular location for sarcomas. Anatomic constraints limit the ability to achieve wide resection margins [1,2], and symptoms usually occur in later stages. Therefore, RPS are frequently overlooked at early stages, and have a worse prognosis and higher local recurrence rates when compared to extremity sarcomas [6,7,10]. Symptoms are commonly associated with displacement, compressive or obstructive phenomena, and include abdominal or back pain, urinary tract obstruction, edema of the lower limbs, nerve compression, and vague digestive symptoms [11,12,13]. From a large study on more than 10,000 soft-tissue sarcoma patients, evidence emerged that early locoregional recurrence has higher rates in RPS and represents a leading cause of death [14]. 

In several series, recurrence rates ranged from 45 to 49% within the first five years and from 60 to 82% at 10 years after initial treatment [10,14,15], followed by a continuous and relentless progression extending out over 20 years [10,13,16]. Lifelong surveillance is therefore advised [10,11,12,13,14,15,16,17,18]. With regards to five-years-overall survival, studies report mortality ranging between 20 and 70% in RPS [1,10,19,20]. While in extremity and visceral sarcoma death is predominantly related to systemic disease, in RPS the dominant cause of cancer-related death is local progression, albeit often with multifocal local progression [14]. Histology has a role in tracing the recurrence pattern: subtypes such as high-grade LMS show a higher risk of developing distant metastasis, rather than local recurrence, after complete resection [1]. Another peculiarity observed in 13% of RPS patients is intraperitoneal dissemination, known as peritoneal sarcomatosis (PS) [21]. Theoretically, RPS should be restricted to the retroperitoneal space. However, sometimes the anatomical boundary is ruptured, either by primary disease or iatrogenically, causing PS, seen as lesions along the peritoneal surfaces or intraperitoneal viscera. PS is related to advanced disease and dismal prognosis [21].

Current guidelines report postsurgical margin as the strongest predictive factor for disease-specific survival and recurrence, as well as histologic subtype and grade [3,18]; however, a recent study by Ekardt et al. [10] demonstrated that these latter parameters are not as significant in 10-years progression-free survival. Size and depth are two other known variables that influence outcomes in sarcomas, especially >5 cm, but in RPS it is truly rare to find lesions <5 cm [9,10,11,12,13,14]. 

In RPS, as well as in other sarcomas, lymph node metastases are rare (<1%) [19]. A combination of lymph node metastasis and other metastases is a poor prognostic sign [19]. 

Lastly, it is important to refer patients with suspected RPS to specialized tertiary referral centers [2,3,18]. Blay et al. [22,23] have shown in two large cohort studies that patients managed in specific reference centers with multidisciplinary tumor board capabilities had significantly better compliance with clinical practice guidelines, better quality of the initial surgery with less reoperations, and lower rates of local and metastatic relapses compared to other patients.

The role of the radiologist is indeed important in RPS diagnosis, management, and follow-up, as the ability to promptly recognize local progression, invasion of nearby structures, and complications has a direct impact on patients’ survival. The rarity and diversity of RPS initial clinical and radiological presentations make them challenging to diagnose. The aim of this review is to present the current state of the art on RPS diagnosis, treatment, and follow-up, presenting the main radiological findings connected with principal RPS subtypes on cross-sectional imaging, providing tips and tricks to help reach an early and correct diagnosis.

## 2. Cross-Sectional Imaging and Differential Diagnosis

### 2.1. Differential Diagnosis Challenges

The detection of retroperitoneal masses includes a wide spectrum of differential diagnoses that must be ruled out, such as metastatic adenocarcinoma, retroperitoneal fibrosis, lymphoma, germ cell tumor, paragangliomas, or Castleman’s disease [7]. The diagnostic pathway firstly includes the identification of the most likely origin of the tumor (e.g., LPS can mimic renal or adrenal angiomyolipoma) [6] by considering clinical and laboratory findings such as patient age (e.g., in young men pediatric tumors and, even if rare in retroperitoneal location, testicular masses [12]) and history (e.g., history of melanoma) and presence of any positive serum markers. If nodal involvement is noted, other diagnosis should be considered upon RPS [12].

Contrast-enhanced computed tomography (CT) is the most useful and widely available first-line imaging technique [1,2,3,4,5,6]. CT allows confirmation of the site and origin of the mass and often offers information on tissue composition (e.g., lipomatous elements, calcifications or myxoid elements, internal necrosis) [12], which are fundamental to understand the possible RPS type and differential diagnosis to consider [13]. While some guidelines on sarcomas affirm that magnetic resonance imaging (MRI) is the main imaging modality in sarcomas of the trunk and that CT has a specific role in calcified lesions, to identify fractures and rule out conditions such as myositis ossificans [2], in RPS CT seems to have a similar performance to MRI [1,18]. On the other hand, ultrasounds and X-rays have a very limited role.

Contrast-enhanced CT of the chest is indicated by the guidelines [18]. Brain MRI should be proposed in alveolar, soft-part sarcoma, clear-cell sarcoma, and angiosarcoma [18,19]. Abdominal or spine MRI is recommended for patients with an iodine contrast allergy, to better assess pelvic involvement, or neuronal foramina tumoral extent, when unclear on CT [2], or when radiotherapy is envisaged, to assess the surrounding edema and local extension [6,7,8,9,10,11,12].

Following appropriate imaging assessment, image-guided percutaneous coaxial core needle biopsy is needed to confirm the diagnosis [2,3,4,11,18]. However, biopsy may underestimate the malignancy grade. Therefore, 18-fluoro-2-deoxy-glucose positron emission tomography/CT (FDG-PET/CT) can be useful, as it carries an excellent diagnostic accuracy (91.8%) in most sarcomas and can help to target, together with diffusion-weighted imaging (DWI), non-necrotic, non-hemorrhagic and viable areas with low apparent diffusion coefficient (ADC) values, with high vascularity and high glucose uptake. PET/CT is also useful to rule out equivocal findings on CT [24,25], and recent studies are also exploring the correlation between SUV max and tumoral grade [25].

Recurrence on imaging may antedate symptomatic recurrence by months to years. Postsurgical imaging follow-up should be performed every 3–6 months for the first 2–3 years, and then annually after five years [26].

With regards to imaging protocols, multiphasic study is recommended, including arterial, portal venous, and delayed phase on CT to better assess local extension, invasion of nearby structures, metastases, or local complications. The relationship with major vessels is a key element in radiological evaluation, as it affects surgery, neoadjuvant treatments, resection, and bypass reconstruction [27]. Sambri et al. [28] recently demonstrated that vascular proximity is an independent predictor of local recurrence. 

Contrast-enhanced MRI study is aimed at tissue characterization. Imaging protocols should include T2- and T1-weighted (w) sequences, on axial and coronal planes, with and without fat suppression, to assess the presence of blood, fluid, gross fat, and fibrotic tissue. DWI and ADC maps are important to depict local and distant extension, and T1-w Dual Echo sequence to assess microscopic fat. Recent studies are exploring the promising role of radiomics in preoperative imaging for pathological prediction [26], assess postradiotherapy changes by using DWI sequences [29], predict prognosis and local recurrence [30,31], and for automatic segmentations with deep learning networks for the automatic tumor volumetric measurements of RPS on CT [32].

### 2.2. Peculiar Imaging Challenges of RPS

All sarcomas on CT have typically a heterogeneous appearance. Sarcoma may be slightly vascularized or early enhancing and can contain necrotic or fluid areas [33,34]. This high variability reflects the polymorphic appearance at histology. RPS grow by direct local extension into adjacent tissues and structures, often pushing them aside or, less commonly, by invading fascial planes or bones [7,33]. Usually, in the case of large masses, RPS may distort normal anatomy, and the displacement of retroperitoneal organs is a useful indicator of a retroperitoneal origin [12]. 

LPS can contain large quantities of gross fat, thus making their extent assessment very difficult [32,33,34]. The most frequent mistakes on RPS imaging are indeed to underestimate the extension of the well-differentiated part of a LPS that presents both a well-differentiated and a dedifferentiated component, and to miss extension across abdominal wall openings such as the inguinal ligament, or extension into the scrotum or adductor compartment of the thigh or posterior extension out of the sciatic notch [7,8,9,10,11]. Considering that LPS represent up to 70% of all RPS, the diagnostic process should start with searching for the presence of abnormal macroscopic fat and RP distortions [6]. Imaging is also challenging in LPS with unclear gross fat, or, in LMS, when an obvious origin from venous structures is not seen. When several organs such as adrenal glands and kidneys are invaded, assessing the origin of the primary tumor is tricky [32]. 

Radiologists should describe tumor location, size in the three-dimensional planes, the relationships and mass effect to other structures and vessels, parietal wall, nervous and vascular structures, and spine, as well as the presence of fat, myxoid, necrotic, and hemorrhagic content [9,33]. Regarding vascular and nervous extension, CT can provide some information on the encasement of arteries, veins, and nerves. With regards to MRI, a study by Holzapfel et al. [35] found a sensitivity of 84.6%, 84.6%, and 77.8%, and specificity of 93.8−97.5%, 94.7, and 97.3%, for T2- and fat-suppressed contrast-enhanced T1-weighted imaging in arteries, veins, and nerves encasement, respectively. It is important to effectively describe vessels involvement since it has many repercussions on both surgery and clinical management. Vascular compression can be seen in all RPS, but some subtypes, such as leiomyosarcoma, are prone to macroscopic vascular invasion or can originate primarily from RP vessels, such as the inferior vena cava (IVC). Leiomyosarcomas arising from the IVC require more specialized surgical treatment than their primary retroperitoneal counterparts [36]. Radiologists should describe each anatomical compartment and structure invaded, aponeurotic spreading, satellite tumors (i.e., multifocality), lymphadenopathy, extension to skin, bone, artery, veins, and nerves. Table 1 includes some of the main differential diagnoses of RPS based on the different internal components that might be seen on imaging.

## 3. Imaging of the Different Subtypes

### 3.1. Liposarcomas

**Figure 1:**
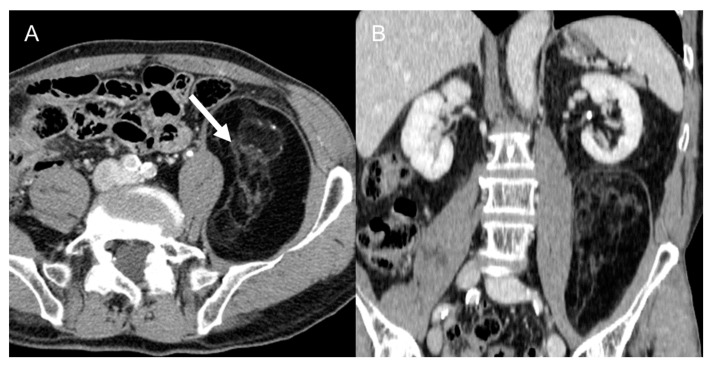
Well-differentiated retroperitoneal liposarcoma in a 71-year-old man. Axial (**A**) and coronal (**B**) contrast-enhanced CT images in the venous phases show a 13.3 cm fat-attenuating mass adjacent to the left psoas muscle, with thin septa (arrow). The lesion was histologically confirmed after surgical excision.

LPS represent the most common primary RPS [2,3,4,5,6,7,8,9,10,11,12,13,14,34], and typically occur in adult patients (50–70 years). LPS commonly present as round, oval, or lobulated intra-abdominal fat-attenuating masses that exert mass effect on adjacent structures. They are histologically subdivided into five different subgroups based on the WHO 2020 classification [8]. LPS with both well-differentiated and dedifferentiated components are often poorly evaluated and described as multifocal masses, because only the dedifferentiated or solid components are demarked, while the well-differentiated fatty mass is often not recognized [37,38]. An incomplete report may lead to incomplete surgery, which worsens the prognosis [11,12]. Lungs are the primary metastatic site [11]. LPS subtypes have some peculiar characteristics that may allow their differentiation, as follows:

A. Well-differentiated liposarcomas (Figure 1) are low-grade tumors. Characteristic CT features include macroscopic fat in at least 75% of the whole tumor with smooth and lobular margins, thick septa (>3 mm), tendency to be nodular, and mild or inconstant low enhancement [5,37,38,39,40]. Calcifications are rare [34] and can indicate dedifferentiation or inflammation. These tumors can recur, but do not tend to metastasize [5].

**Figure 2:**
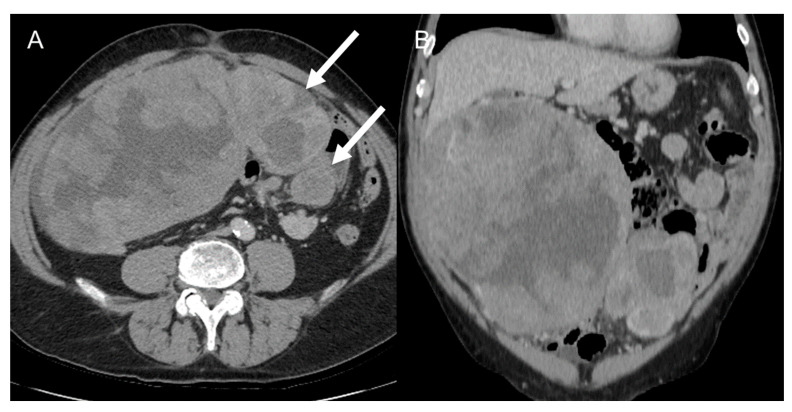
Dedifferentiated retroperitoneal liposarcoma in a 68-year-old man. Axial (**A**) and coronal (**B**) contrast-enhanced CT images in the venous phases show a 24 cm solid mass in the right retroperitoneal space, with internal necrotic areas and adjacent nodules (arrows). The lesion was histologically confirmed at biopsy.

B. Myxoid/Round-cell liposarcomas (MLS) are intermediate-grade tumors and almost always occur in the retroperitoneum as secondary locations [41]. They are heterogeneous, lobular, with internal septations and features often described as “pseudocystic” due to myxoid components. Compared to true cystic lesions, they gradually enhance on delayed postcontrast phases, with progressive accumulation of contrast within the myxoid matrix [37,38]. In more than 50% of cases, there is no fat component [5,37]. Calcifications are rare. Unlike other sarcoma subtypes, it has a propensity for pulmonary, and extrapulmonary metastases, that do not uptake contrast on FDG-PET [41]. Of note, there is a preponderance of spinal metastases, not clearly visible on CT. Clinical practice guidelines have therefore included spine MRI as part of MLS staging [42].
C. Dedifferentiated liposarcomas (Figure 2) are high-grade tumors with poor prognosis. Characteristic features include heterogeneous nonlipomatous mass within, adjacent to, or surrounding a fatty mass [37,38]. There may be no evidence of fat-density tissue in up to 20% of cases, making the imaging diagnosis difficult [40]. Enhancing septa within the fatty portions are frequently seen [37]. Calcifications are rare (around 25% of cases) and are poor prognostic factors [39].

D. Pleomorphic liposarcomas contain little or no fat and myxoid components. They are considered high-grade malignancies with high rates of local recurrence and distant metastases [38]. They are heterogeneous masses, isoattenuating to muscles on CT and commonly have internal areas of low attenuation representing necrosis. Calcifications are rare [12].
E. Undifferentiated pleomorphic liposarcoma: imaging features are nonspecific. It manifests as a large, well-circumscribed soft-tissue mass with heterogeneous enhancement and myxoid components. Areas of necrosis and hemorrhage may be seen but are less extensive than leiomyosarcomas. Calcifications occur in up to 20% of cases with a ring-like pattern. Direct invasion of adjacent organs may be present [37].

**Figure 3:**
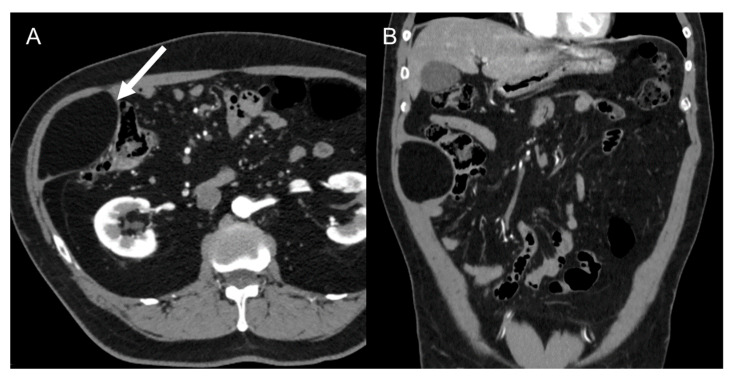
Abdominal lipoma in a 67-year-old man. Axial (**A**) and coronal (**B**) contrast-enhanced CT images in the venous phases show a 6.8 cm fatty mass (arrow) in the right abdominal wall, with no internal septa or nodules.

The main differential diagnosis is with simple lipomas (Figure 3) that will present as purely adipocytic tumors [38]. In this case, when the lesion is ≤10 cm, the patient can be managed without a biopsy. Beyond 10 cm, patients should still undergo MRI and biopsy [9]. Another diagnosis to keep in mind is renal angiomyolipoma, which, contrary to LPS, is hypervascular and presents with a large vessel extending into the renal cortex. Moreover, the presence of a renal parenchymal defect at the site of tumor contact favors exophytic angiomyolipoma. Other rarer occurrences to consider are adrenal myelolipomas, which share similar imaging appearance with angiomyolipoma, and ovarian teratomas. Features that should favor the latter are the presence of fat–fluid levels or tooth-like calcifications [38].

After surgery, retroperitoneal LPS have a high tendency to recur locally, while metastases are less common. Intraoperatively, low-grade LPS is grossly like normal fat, thus making the resection challenging, as frozen section evaluation is considered unhelpful [2]. Preoperative resection planning is guided by asymmetry shown on imaging, knowledge of functional anatomy, and experience with patterns of recurrence [2].

### 3.2. Leiomyosarcoma

**Figure 4:**
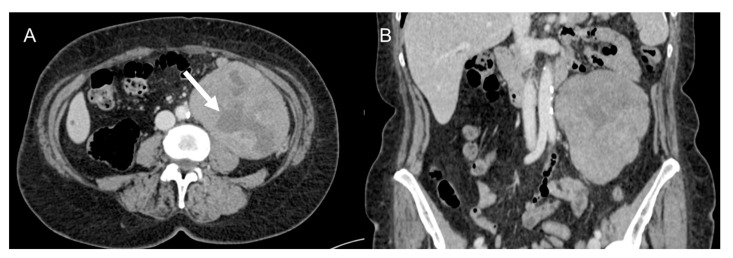
Retroperitoneal leiomyosarcoma in a 63-year-old woman. Axial (**A**) and coronal (**B**) CT images on venous phase show a 10.2 cm heterogeneous mass with central area of low attenuation consistent with necrosis (arrow).

LMS is a smooth muscle tumor [38], more common than LPS in younger age groups [6]. Its most frequent location is the retroperitoneum, near the inferior vena cava [43]. LMS are heterogeneous masses (Figure 4), with irregular peripheral enhancement and enhancing solid portions, mixed with cystic, hemorrhagic, or necrotic areas. LMS show marked T2 hypointensity on MRI and are similar in attenuation to uterine myometrial smooth muscle on CT [39]. Fatty components and calcifications are usually absent [34,38]. The evidence of a large, heterogeneously enhancing, necrotic retroperitoneal mass contiguous with a vessel, with extra- and intraluminal involvement, is highly suggestive of LMS [12,37,43]. It could stem from the inferior vena cava, extending into the intrahepatic portal veins or superior mesenteric vein, or even from small vessels such as renal or gonadic veins. Differentiation from extrinsic compression can be challenging [12,40]. 

### 3.3. Solitary Fibrous Tumors (SFT)

**Figure 5:**
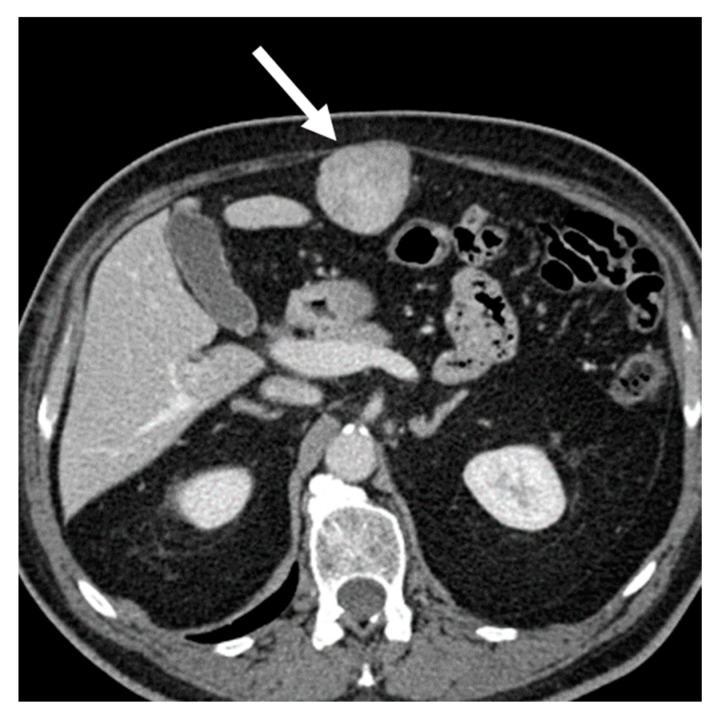
Solitary fibrous tumor in 76-year-old man. Axial CT on venous phase shows a 5 cm well-defined solid mass with heterogeneous enhancement (arrow). The lesion was histologically confirmed at biopsy.

Although their imaging appearance is nonspecific, most solitary fibrous tumors (Figure 5) show heterogeneity and are highly vascular, with prominent collateral vessels [38,44,45]. These tumors are well defined, with intense delayed contrast enhancement due to the fibrous stroma [40]. SFT may rarely exhibit cystic degeneration, necrosis, and calcifications [44]. MRI signal intensity is variable and dependent on cellularity and the abundance of myxoid stroma and/or fibrous tissue [40]. Solitary fibrous tumors are typically hypo-to-isointense to muscle on T1-weighted MRI and hypointense on T2-weighted sequences [45]. Flow voids may be present because of prominent intralesional vessels [45,46].

### 3.4. Nerve Sheath Tumors (NST)

**Figure 6:**
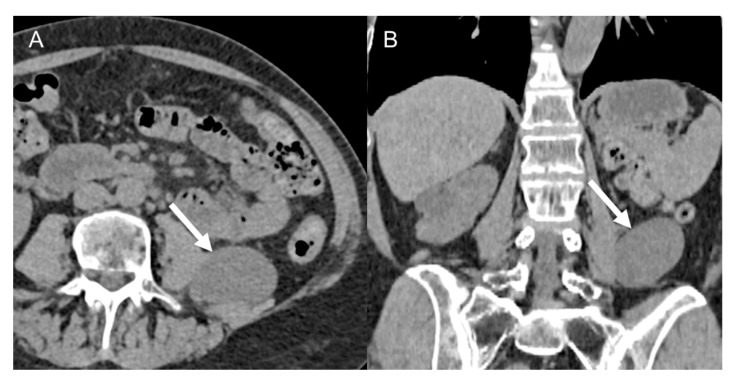
Retroperitoneal schwannoma in 66-year-old woman. Axial (**A**) and coronal (**B**) non-contrast CT images show 5.4 cm homogeneously hypodense lesions with posteriorly to the right psoas muscle (arrow).

In the retroperitoneum, a mass originating proximally to the spine, in the psoas muscle, in the sciatic nerve, or sacral plexus, or involving the neural foramina (which must be checked) can suggest a neural origin [11,47]. Schwannoma is the most frequent benign tumor (Figure 6), while MPNST is the most frequent malignant one [11]. Benign sporadic schwannomas have an unknown etiology, but if multiple lesions are seen, neurofibromatosis should always be ruled out, as it is associated with a higher risk of malignant degeneration [12,34,47]. 

Benign NST are usually rounded and well defined, with subtle, homogeneous enhancement, but the differential diagnosis with MPNST is oftentimes not doable on imaging [11,40]. The presence of ill-defined, infiltrative, margins, inhomogeneous peripheral enhancement, osseous involvement or encasement of nearby structures and vessels, sudden fast growth and pain are all factors pointing towards MPNST [11,12,34,35]. MPNST MRI signal is inhomogeneous, with T1 isointensity and T2 hyperintensity, cystic degeneration and lack of capsulation [40]. 

Table 2 summarizes the types of RPS described in this section.

## 4. How to Diagnose RPS on Imaging

### 4.1. Tips and Tricks: CT

**Figure 7:**
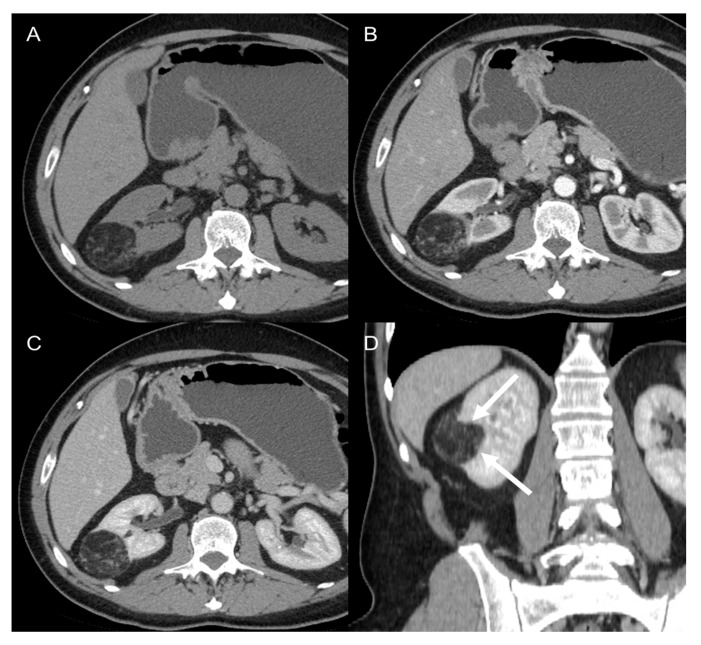
Renal angiomyolipoma in 62-year-old man. Axial CT images on the pre-contrast (**A**), arterial (**B**), and venous (**C**) phases and coronal venous (**D**) phase show a 4.5 cm heterogenous adipose lesion deforming the edge of the right kidney with a “beak” shape appearance (claw sign, arrows).

**Figure 8:**
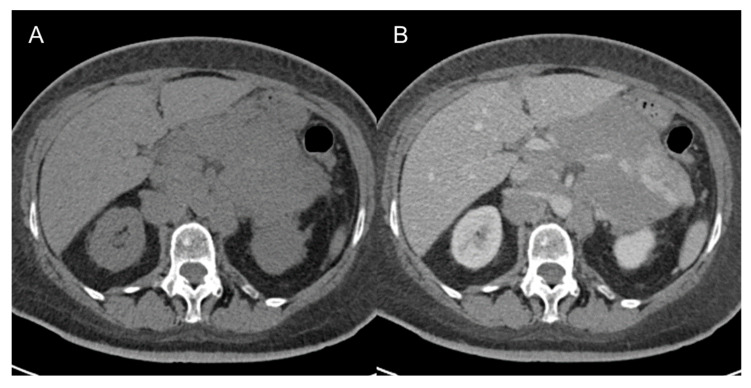
Non-Hodgkin lymphoma in 62-year-old man. Axial CT images on the pre-contrast (**A**) and venous (**B**) phases show multiple confluent retroperitoneal and mesenteric nodal masses on both sides of the upper abdominal vessels (sandwich sign).

Some CT features can help to identify the organ of origin of RPS:

- The beak (claw) sign: when a mass deforms the edge of an adjacent organ into a “beak” shape, it is likely that the mass arises from that organ (e.g., a notch or an infiltration of the renal hilum, suggests a kidney origin of the tumor in the differential diagnosis between LPS and angiomyolipoma (Figure 7) [11,46]).
- The embedded organ sign: when there is intimate contact between the mass and the organ of origin, a desmoplastic reaction and sometimes ulcerations are observed (positive sign). On the contrary, a moldable, compressed organ will be deformed into a crescent shape (negative sign). The latter is useful for example in the differential diagnosis between RP leiomyosarcoma and primitive inferior cava leiomyosarcoma [36,46].
- The phantom or invisible organ sign: if an organ cannot be seen, it is probably the origin of the mass. This sign can lead to false positives diagnoses, as large sarcomas can invade the adrenal gland.
- Prominent feeding artery sign: hypervascular masses in most cases are supplied by feeding prominent arterial vessel that can be studied on CT and can help to understand the origin of the mass (e.g., identifying a prominent vessel originating from the kidney balances the diagnosis towards angiomyolipoma).
- The sandwich (or hamburger) sign (Figure 8): presence of a mesenteric nodal mass that envelops mesenteric vessels on both sides, without grossly infiltrating them. This is a classic sign of mesenteric lymphoma (typically non-Hodgkin) or posttransplant lymphoproliferative disorder in transplanted patients.

CT is also useful to identify patterns of growth and dissemination, which is fundamental for surgical and treatment planning in multidisciplinary contexts, and useful to identify the sarcoma subtype. Lesions surrounding normal structures and vessels without invading or compressing them and affecting more than one compartment will more likely be lymphangiomas and lymphomas; conversely, lesions that are located along the sympathetic chain will most likely be paragangliomas or ganglioneuromas.

The assessment of the enhancement pattern is another fundamental aspect to search on CT to understand the nature of a lesion, as follows:

- Absence of enhancement is commonly seen in cysts, hematomas, and simple lipomas.
- Early enhancement and rapid washout is seen in paragangliomas and Castleman’s disease.
- Early enhancement and delayed or imperceptible washout is unspecific, as it is seen in most tumors (benign or malignant), such as paragangliomas.
- Late enhancement is commonly seen in neurogenic tumors, desmoids, leiomyomas, leiomyosarcoma, and lymphoma.

Lastly, CT can also help to identify tumoral components such as fat, myxoid stroma, necrosis, cystic degeneration, and calcifications. Calcifications can be present and indicate dedifferentiation and poor prognosis or sclerosing or inflammatory variants of well-differentiated LPS [6].

### 4.2. Tips and Tricks: MRI

**Figure 9:**
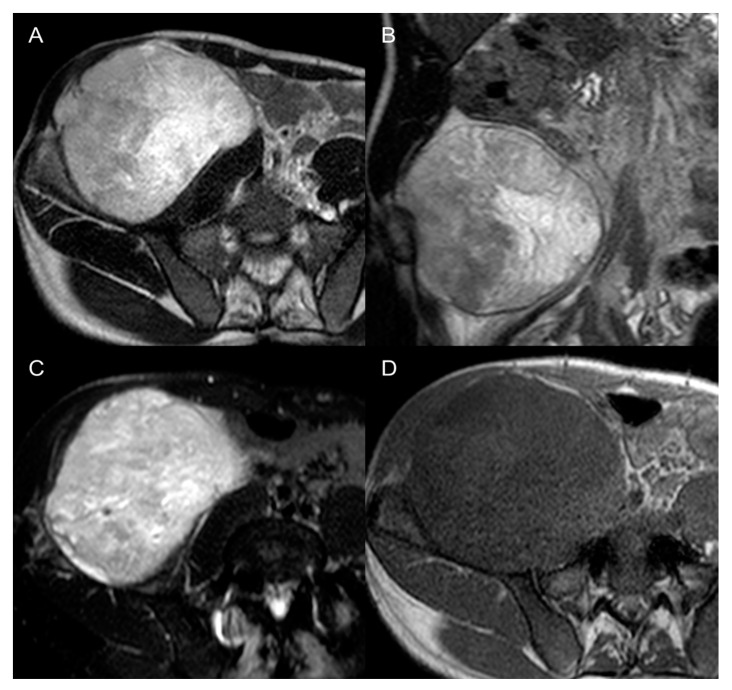
Pleomorphic rhabdomyosarcomas in a 37-year-old man. Axial (**A**) and coronal (**B**) T2-weighted MR images, axial SPAIR (**C**), and axial T1 GRE (**D**) images show a 12 cm large heterogenous mass in the right retroperitoneum. The lesion was histologically confirmed at biopsy.

Contrast-enhanced MRI is the best modality to characterize a soft-tissue tumor, but also for local staging and prognosis. The diagnostic orientation can be facilitated on MRI by the identification of myxoid stroma (high fluid-like signal on T2, hypointense on T1, with contrast uptake), fatty content (Dixon [9] and Dual Echo), necrosis, hemorrhage, cystic degeneration, and fibrotic content. For instance, the presence of myxoid stroma can point towards myxoid round cells LPS, low-grade fibromyxoid chondrosarcoma, rhabdomyosarcoma (Figure 9), or small extraskeletal myxoid chondrosarcoma [9]. Undifferentiated sarcoma, instead, can present as massively hemorrhagic tumors, therefore being similar to a large hematoma, that does not have an explanation, nor a rapid appearance or resolution [6,9].

On MRI, in peripheral NST, specific signs such as the target sign or the split fat sign and the entering nerve sign can be seen [9,34]. In all RPS, when infiltrative patterns are present, wider margins of resection should be obtained, as they are predictive of grade III tumors; therefore, the radiologist must acknowledge them [9]. Other elements indicative of a grade II–III tumor is high necrotic content, peritumoral edema, or peritumoral enhancement [9].

**Figure 10:**
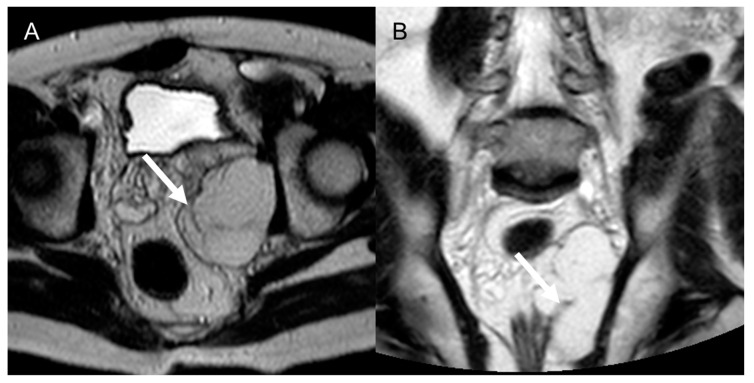
Dedifferentiated retroperitoneal liposarcoma in a 50-year-old man. Axial (**A**) and coronal (**B**) T2-weighted MR images show an 8 cm fatty mass in the felt pelvis, with internal septa (arrows). The lesion was histologically confirmed after surgical excision.

In myxofibrosarcoma and myxoid LPS, the water-like sign on T2-w sequences is related to high myxoid matrix content (≥75%) and has been associated with an increased risk of local recurrence and worse prognosis [48], as it is associated with shorter metastasis-free survival [27]. A higher amount (≥50%) of fatty content also seemed to correlate with higher grade and shorter metastasis-free survival [27] (Figure 10).

In LMS, instead, a prognostic factor is the quantification of necrosis, as higher necrosis is associated with higher grade of the disease [43].

In all RPS, evidence of osseous invasion is linked to lower disease-free survival and overall survival, while vascular or nerve invasion and multifocality are associated with higher local recurrence rates. Nodal invasion instead correlates also with higher probability of metastases [9,35].

MRI helps also in the differential diagnosis with lymphoma facing a hypointense T1 homogeneous mass with a homogeneous enhancement and strong diffusion restriction, without necrotic, blood, fibrotic or fat signal, no peritumoral enhancement or abnormal intra- and peritumoral vasculature, and a possible infiltrative growth pattern [9].

## 5. Complications

**Figure 11:**
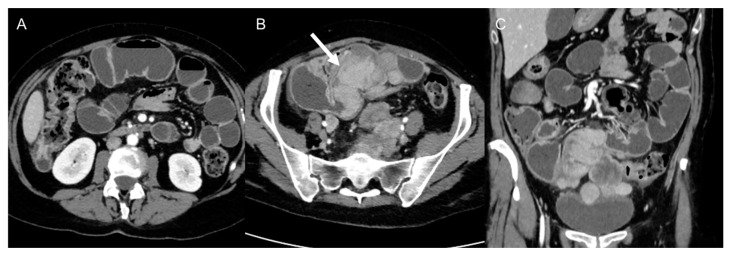
Bowel obstruction in 38-year-old woman with metastatic leiomyosarcoma. Axial (**A**,**B**) and coronal (**C**) CT images on the venous phase show dilatation of small bowel loops with air–fluid levels secondary to the presence of metastatic masses (arrow) in the pelvis.

Complications are usually related to tumor invasion of adjacent structures or to mass effect caused by the tumor and include hydronephrosis, pulmonary embolisms secondary to compression of inferior cava vein, nerve compression, bowel obstruction (Figure 11) [49], and even intestinal perforation [50]. Postoperative complications are divided into early and late complications and include pulmonary embolism, ileus, fluid collections, hemorrhage [49], splenic injuries, sepsis, multisystem organ failure. For these reasons, guidelines recommend that immediate postsurgical care should be held in subspecialized facilities [2,19,51,52,53].

## 6. Treatment Options and the Role of the Radiologist

An optimal management of RPS is facilitated by a comprehensive pretreatment diagnosis and staging, for which the radiologist has a primary role. Pretreatment pathologic diagnosis is needed, but complete surgical resection with negative margins is the cornerstone for nonmetastatic RPS and is the only proved curative strategy. This is achieved by en bloc resection of the tumor and all surrounding structures, even if not overtly infiltrated on imaging or surgical exploration [2,7,11,17,18,51], with the aim of obtaining R0 margins; however, this goal is difficult, especially in nonspecialized centers. Since patients with R1 margins tend to have significantly lower mid-term survival rates and higher recurrence rates [52,53,54,55], but at the same time a far too demolitive surgery can have a certain impact on a patient’s life, the optimal extent of resection is still debated and considers several factors, such as histological subtype, preoperative therapies, or anatomical barriers. Novel surgical techniques are being adopted to improve radical excision [52,53]. Current guidelines recommend referring patients to multidisciplinary discussion and decision making with radiologists, surgeons, and oncologists who have consolidated experience in the management of soft-tissue sarcomas and in specialized centers [2,11,12,18,54,55,56]. 

In cases of recurrence, surgery should be aimed at achieving macroscopic complete resection, including surrounding organs only when overtly infiltrated [2]. Grossly incomplete resections are questionable and potentially harmful but could be planned out as palliative surgery in selected patients [2].

Currently, there is no evidence to support the routine use of adjuvant radiation therapy (RT) or adjuvant chemotherapy in RPS [56]. Neoadjuvant RT has been prospectively evaluated in a recent randomized controlled trial (STRASS) [51], suggesting an improved abdominal recurrence-free survival only in well-differentiated LPS, and lack of effect for LMS or high-grade dedifferentiated LPS, since these two subtypes have a predominant pattern of distant rather than local progression [18]. This evidence has prompted new studies on neoadjuvant chemotherapy (EORTC1809-STRASS2), targeted for these histological subtypes [1,3,18]. Future evidence may emerge from novel systemic therapy options with targeted and immunotherapeutic therapies. 

## 7. Conclusions

Radiologists play a primary role in preoperative diagnosis and staging of RPS, detection of recurrence, and assessment of postoperative complications. Many imaging patterns can overlap among the various subtypes of sarcomas. Nonetheless, some specific imaging appearances can guide the radiologist. This is of the utmost importance, as imaging has a central place in each phase of the management of patients, and both nonspecialized and specialized radiologists can have a strong impact in the management and survival of patients with RPS. Indeed, the radiologist is commonly among the first physicians to raise the suspicion of the diagnosis of sarcoma. Imaging features with patterns of involvement, associated to clinical features and a multidisciplinary approach through discussions in tumor board, is fundamental.

## Figures and Tables

**Table 1:** CT imaging appearance of common retroperitoneal sarcomas based on internal component with differential diagnosis.

Components	Tumor	Imaging Features	Differential Diagnosis
Fat	- Lipoma	- Well defined - Mainly fat component - Lacking fibrous strands, septations, or solid components - No enhancement	- Lipomatosis - Fat Necrosis (to consider if surgery) - Germ Cell tumors (especially younger patients) - Extramedullary hematopoiesis (investigate hematological history and laboratory data) - Exophytic giant renal angiomyolipoma (if renal origin) - Metastases (rare, only if primitive tumor contains fat) - Pancreatic Lipomatosis - Giant adrenal myelolipoma (if adrenal origin) - Hibernoma (very rare in retroperitoneum) - Ovarian teratoma (if ovarian origin)
	- Liposarcoma:	- Different imaging appearance according to subtype	
	- Well differentiated	- Predominantly fat-containing lesions with minimal soft-tissue parts	
	- Myxoid	- Pseudocystic appearance due to the large amounts of extracellular myxoid material - Thick septa and patchy or nodular soft-tissue parts	
	- Dedifferentiated	- Heterogeneous mass with fat components - Calcifications (rare) - Enhancing septa	
	- Pleomorphic - Round cell	- Minimal—if any—visible fat - Attenuation and signal intensity approximate those of adjacent muscle	
Calcifications			
	- Dedifferentiated Liposarcoma	- Heterogeneous fatty mass - Enhancing septa	- Germ Cell tumors (younger patients) - Myelolipoma (adrenal >> retroperitoneal) - Hemangiopericytoma (high arterial enhancement) - Neurogenic Tumors (neural foramina, neural cells) - Ovarian teratoma (if ovarian origin) - Uterine fibroids (if uterine)
	- Undifferentiated pleomorphic liposarcoma	- Large and lobulated - Dystrophic calcifications (25%), uncommon amongst the remaining primary retroperitoneal malignancies - Center of the lesion with lower attenuation (necrotic/cystic changes)	
	- Chondrosarcoma	- Large soft-tissue mass - Chondroid ring or arc type of calcifications or amorphous punctate calcifications	
	- Ewing sarcoma	- Well-defined nonspecific soft-tissue mass of similar attenuation to muscle - Calcification - If osseous involvement of bone surface, there is cortical erosion or periosteal reaction	
Cystic			
	- Myxoid liposarcomas	- Thick septa and patchy or nodular soft-tissue parts	- Post-chemotherapy cystic changes in solid neoplasms (check previous imaging) - Lymphangioma (younger patients) - Mucinous/serous cystadenoma (if pancreatic) - Teratoma (multiple components, including fat) - Cystic mesothelioma (exposure to asbestos, ill-defined infiltrative margins) - Müllerian cyst (males, anywhere along this path of Müllerian duct regression) - Epidermoid cyst (homogeneously fluid) - Tailgut cyst (septated cysts, from vestiges of the embryonic hindgut) - Bronchogenic cyst (left adrenal region and the superior body of the pancreas) - Hematoma (subacute trauma) - Pseudomyxoma retroperitonei (scalloped appearance of abdominal organs and omental caking +/− appendiceal mucocele) - Pancreatic pseudocyst (if pancreatitis history) - Lymphocele (if recent surgery) - Urinoma (if urinary obstruction, iatrogenic lesion)
	- Undifferentiated pleomorphic liposarcoma	- Lobulated - Central necrotic/cystic changes	

**Table 2:** Main CT imaging appearance of common retroperitoneal sarcomas.

Type	Main Characteristics
Liposarcomas	Most common overall Variable fat component 5 histological subtypes
I. Well-differentiated liposarcomas	Fat ≥ 75% Smooth margin, septa, mild or inconstant enhancement Recurrence, but no metastases
II. Myxoid/Round-cell liposarcomas (MLS)	Intermediate grade +myxoid component (appearing as cystic on) +/− fat Recurrence and metastasis
III. Dedifferentiated liposarcomas	High grade, poor prognosis Heterogeneous non-fat mass + fat mass Enhancing septa in fatty portions Calcifications (25%)
IV. Pleomorphic liposarcomas	High grade, high rates of recurrence and metastases Heterogeneous masses, isoattenuating to muscles, +/− necrosis
V. Undifferentiated pleomorphic liposarcoma:	Nonspecific imaging Large heterogeneous masses Invasion of neighbor organs
Leiomyosarcoma	Most common in younger patients Heterogeneous solid masses Peripheral enhancement Cystic, hemorrhagic, or necrotic areas NO fat or calcifications
Solitary Fibrous Tumors (SFT)	Highly vascular Prominent collateral vessels Intense delayed enhancement (fibrous stroma)
Nerve sheath tumors (NST)	Close to spine, psoas muscle, sciatic nerve, sacral plexus, other neural foramina Benign if well-defined, small, well-rounded, subtle homogeneous enhancement Malignant if infiltrative margins, peripheral enhancement, encasement of nearby structures
